# A novel diagnostic four-gene signature for hepatocellular carcinoma based on artificial neural network: Development, validation, and drug screening

**DOI:** 10.3389/fgene.2022.942166

**Published:** 2022-09-28

**Authors:** Min Chen, Guang-Bo Wu, Zhi-Wen Xie, Dan-Li Shi, Meng Luo

**Affiliations:** ^1^ Department of General Surgery, Shanghai Ninth People’s Hospital Affiliated to Shanghai Jiao Tong University School of Medicine, Shanghai, China; ^2^ Department of Urology, Shanghai General Hospital, Shanghai Jiao Tong University School of Medicine, Shanghai, China

**Keywords:** hepatocellular carcinoma, diagnostic model, artificial neural network, MT1M, SPINK1, AKR1B10, SLCO1B3

## Abstract

**Background:** Hepatocellular carcinoma (HCC) is one of the most common cancers with high mortality in the world. HCC screening and diagnostic models are becoming effective strategies to reduce mortality and improve the overall survival (OS) of patients. Here, we expected to establish an effective novel diagnostic model based on new genes and explore potential drugs for HCC therapy.

**Methods:** The gene expression data of HCC and normal samples (GSE14811, GSE60502, GSE84402, GSE101685, GSE102079, GSE113996, and GSE45436) were downloaded from the Gene Expression Omnibus (GEO) dataset. Bioinformatics analysis was performed to distinguish two differentially expressed genes (DEGs), diagnostic candidate genes, and functional enrichment pathways. QRT-PCR was used to validate the expression of diagnostic candidate genes. A diagnostic model based on candidate genes was established by an artificial neural network (ANN). Drug sensitivity analysis was used to explore potential drugs for HCC. CCK-8 assay was used to detect the viability of HepG2 under various presentative chemotherapy drugs.

**Results:** There were 82 DEGs in cancer tissues compared to normal tissue. Protein–protein interaction (PPI), Gene Ontology (GO), and Kyoto Encyclopedia of Genes and Genomes (KEGG) enrichment analyses and infiltrating immune cell analysis were administered and analyzed. Diagnostic-related genes of *MT1M*, *SPINK1*, *AKR1B10*, and *SLCO1B3* were selected from DEGs and used to construct a diagnostic model. The receiver operating characteristic (ROC) curves were 0.910 and 0.953 in the training and testing cohorts, respectively. Potential drugs, including vemurafenib, LOXO-101, dabrafenib, selumetinib, Arry-162, and NMS-E628, were found as well. Vemurafenib, dabrafenib, and selumetinib were observed to significantly affect HepG2 cell viability.

**Conclusion:** The diagnostic model based on the four diagnostic-related genes by the ANN could provide predictive significance for diagnosis of HCC patients, which would be worthy of clinical application. Also, potential chemotherapy drugs might be effective for HCC therapy.

## Introduction

Hepatocellular carcinoma (HCC) is an increasingly serious public health problem, and it is gradually becoming one of the main causes of cancer mortality in the world (Villanueva, 2019). As most cases of HCC occur in patients with underlying chronic liver diseases like hepatitis B virus (HBV) infection and varying degrees of cirrhosis, the diagnosis of HCC in humans is challenging (Vogel and Saborowski, 2020; Yang and Heimbach, 2020). Most HCC patients are diagnosed when they have obvious clinical symptoms appear or are at advanced stages of the disease, which reduces or even precludes the effective use of curative therapy (Ayuso et al., 2018; Rastogi, 2018). Therefore, identifying candidate biomarkers and constructing novel diagnostic models could be useful in distinguishing HCC patients from normal people, which would improve the overall survival (OS) of HCC patients.

In the clinic, the detection of serum tumor markers has been widely used for its advantages of the noninvasive method (Han et al., 2020). However, the information provided by the conventional assays for carcinoembryonic antigen (CEA) and carbohydrate antigen 19-9 (CA19-9) is not specific or sensitive enough (Cui et al., 2016; Edoo et al., 2019). Thus, developing novel diagnostic biomarkers is necessary for early detection of HCC. Meanwhile, HCC patients could not get timely treatment because of multidrug resistance or might have suffered from severe drug-related adverse effects from chemotherapy (Zhang et al., 2016). Calling for novel and effective drugs for HCC patients is an eternal theme of the times.

With the improvement of bioinformatics technology, differentially expressed genes (DEGs) by systematic bioinformatics analysis could be employed to select candidate genes and underlying pathways that were related to the occurrence and progression of HCC for diagnosis (Wang et al., 2018; Li et al., 2021). Moreover, an artificial neural network (ANN) is a classic machine learning method, which is often used for modeling construction (Zhong et al., 2019; Mai et al., 2020). In this study, we first retrieved transcriptional expression data of patients with HCC patients from GEO datasets and found 82 DEGs between normal and HCC tissues. Next, the possible functional mechanisms were explored by protein–protein interaction (PPI), Kyoto Encyclopedia of Genes and Genomes (KEGG), and Gene Ontology (GO) enrichment analyses. Then, metallothionein 1M (MT1M)*,* solute carrier organic anion transporter family member 1B3 (SLCO1B3), serine protease inhibitor Kazal type 1 gene (SPINK1), and aldo–keto reductase family 1B10 (AKR1B10) were found and used as candidate biomarkers to construct an artificial neural network (ANN) model. Further validation of diagnostic-related genes was performed by QRT-PCR in HepG2 and HL7702 cell lines. The potential drug for HCC therapy, including vemurafenib, LOXO-101, dabrafenib, selumetinib, Arry-162, and NMS-E628, were found based on four diagnostic-related genes. Vemurafenib, dabrafenib, and selumetinib might have a broad application prospect in HCC.

## Methods

### Datasets

The RNA microarray data of HCC samples and corresponding normal liver tissue were retrieved from the GEO dataset (https://www.ncbi.nlm.nih.gov/geo/). The datasets of GSE14811, GSE60502, GSE84402, GSE101685, GSE102079, GSE113996, and GSE45436 were downloaded and divided into training and testing cohorts. The “sva” package of R was used to ensure that the GEO datasets were batch effects-corrected before bioinformatics analysis to avoid generating less reliable results (Leek et al., 2012). The first six datasets were classified as training cohorts and used for diagnostic model construction. The latter was classified as a testing cohort and used for validation.

### Identification of differentially expressed genes

Normalization of the count matrix was performed with the trimmed mean of the M-values normalization method of the edgeR (R package). The limma R package was used to identify differentially expressed genes (DEGs) in the construction cohort. The screening standards of DEGs for functional enrichment analysis were |log_2_FC|> 1 and FDR<0.05. The screening standards of DEGs for diagnostic-related genes used for ANN model establishment and drug-sensitive analysis were |log_2_FC|> 2 and FDR<0.05.

### Functional enrichment analysis

For protein–protein interaction (PPI), we used the String (Protein–Protein Interaction Networks, V: 10.5) database (https://string-db.org/). Kyoto Encyclopedia of Genes and Genomes (KEGG) and Gene Ontology (GO) enrichment analyses of the DEGs were performed by using the R clusterProfiler package, including the packages of “GOplot,” “ggplot2,” “stringi,” “colorspace,” and “digest”. Then, the pathway and process enrichment analyses were carried out using Metascape (Metascape, http://metascape.org). As for infiltrating immune cell analysis, the R package of “e1071,” “corrplot,” and “vioplot” were used for analysis and depicting differences. The 22 representative immune cells and gene expressions in every kind of immune cell to distinguish immune cells from each other are shown in Supplementary Table S1.

### Construction and validation of the diagnostic model

The artificial neural network (ANN) classifier was a feed-forward neural network with three layers, which included input nodes, a hidden layer, and output nodes. The multi-layer perceptron method was incorporated, and training of the network was based on the feed-forward back propagation method to adjust the internal factors of the network, which reduced the overall errors during the repeated development cycles. The ANN model learned to connect the relations between the input and output layers by adjusting the weights and biases of the hidden layer. Here, we used the training cohort (GSE14811, GSE60502, GSE84402, GSE101685, GSE102079, and GSE113996) to establish the ANN diagnostic model, and the testing cohort (GSE45436) was used as the validation one. There were 221 normal tissues and 284 HCC tissues in the training cohort, while 41 normal tissues and 93 HCC tissues were in the testing cohort.

### Cells and cell culture

The HepG2 cell line (Human HCC) and HL7702 (human normal cells) were purchased from the Chinese Academy of Medical Sciences (Beijing, China). The HepG2 cells were grown in Dulbecco’s modified Eagle’s medium (DMEM) supplemented with 10% fetal bovine serum (Life Technologies, Inc., Carlsbad, CA, United States), 1 mM sodium pyruvate, 0.1 mM non-essential amino acids, and 2 mM l-glutamine at 37°C and in 5% CO_2_ in a humidified incubator. The HL7702 cells were cultured in RPMI-1640 medium (Gibco, Rockville, MD, United States) supplemented with 10% Fetal Bovine Serum (Life Technologies, Inc., Carlsbad, CA, United States) at 37°C in a humidified 5% CO_2_ atmosphere.

### Quantitative real-time PCR

Here, we used HepG2 and HL7702 to verify the expression of diagnostic genes. The total RNA was isolated by TRIzol reagent (Life Technologies Corporation, Carlsbad, CA, United States) under the manufacturer’s s directions. Then, 0.8 μg mRNA was used for synthesis of 20 μL cDNA using Superscript II reverse transcriptase and random hexamers (Invitrogen, Carlsbad, CA, United States). Q-RT PCR was further performed on an ABI PRISM 7300 Sequence Detection System with SYBR Green PCR Master Mix (Applied Biosystems). The primers used in this study were *MT1M* (forward 5′-ATT​GAA​TTC​GGA​TGG​ACC​CCA​ACT​GCT​C-3′, reverse 5′-ATT​CTC​GAG​TCA​GGC​ACA​GCA​GCT​G-3′), *SLCO1B3*(forward 5′-TCA​TAA​ACT​CTT​TGT​TCT​CTG​CAA-3′, reverse 5′-GTT​GGC​AGG​CAT​TGT​CTT​G-3′), *SPINK1* (forward 5′-AAC​ACT​GGA​GCT​GAC​TCC​CT-3′, reverse 5′-ATC​AGT​CCC​ACA​GAC​AGG​GT-3′), and *AKR1B10* (forward 5′-CAT​ATC​CAG​AGG​AAT​GTG​ATT​GTC​A-3′, reverse 5′- AGA​CCT​GAA​TGT​TCT​CAA​CAA​TGC-3′). GAPDH was the internal comparison. The mRNA expression of relative genes was calculated using the 2−ΔΔCt method with normalization to GAPDH expression.

### Drug-sensitive analysis of the gene signatures

The transcriptional expression of NCI-60 human cancer cell lines was downloaded from the CellMiner project page (https://discover.nci.nih.gov/cellminer). Pearson’s correlation analysis was performed to determine the association between diagnostic genes and drug sensitivity.

### Cell counting kit-8 (CCK-8 assay)

CCK-8 assay was used to detect the viability of HepG2 under various presentative chemotherapy drugs. The cells were resuspended, seeded in a 96-well plate (6 × 104 cells/well), cultured at an appropriate environment (37°C, 5% CO_2_.), and continually incubated for 2 h with 10 ul CCK-8 solution (Yeasen, Shanghai, China) added to each well. The absorbance of each well was measured at 450 nm and tested by a microplate reader (Bio-Rad, Hercules, CA). The calculation of cell viability was processed as follows: (Experimental group - blank control)/(Negative control - blank control) ×100%. The blank groups contained DMEM medium only, while the negative groups were set up with HepG2 and HL7702 cultured in DMEM-F12 without drugs. Vemurafenib (S1267) and Selumetinib (S1008) were obtained from Selleck Chemicals (Houston, TX, United States). Dabrafenib was purchased from Merck (Kenilworth, NJ, United States). Binimetinib and larotrectinib were provided by Med Chem Express (Shanghai, China).

## Results

### Identification of DEGs

**FIGURE1:**
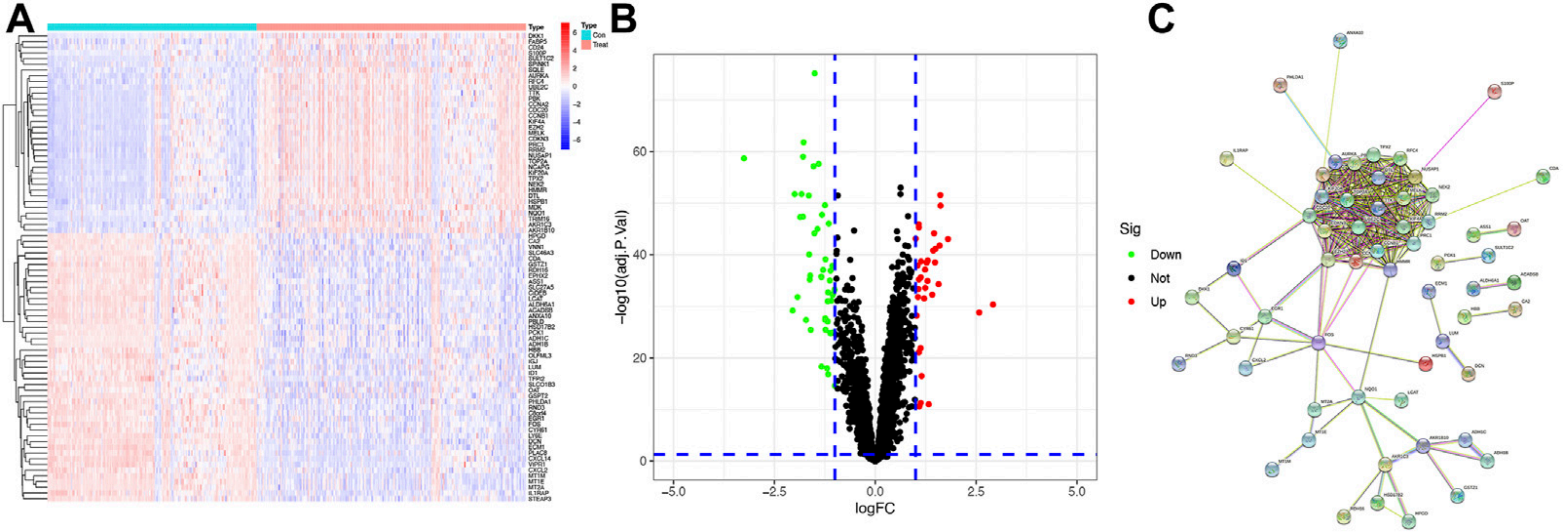
Identification of candidate genes **(A)** Heatmap of differential gene expression between normal and HCC samples in the training cohort, log_2_FC = 1. **(B)** Volcano map of differential gene expression between normal and HCC samples in the training cohort. **(C)** PPI network between these genes (cutoff = 0.4).

A total of 221 normal samples and 284 tumor samples obtained from the GEO dataset formed the training cohort and participated in the identification of DEGs. The differential expression of 82 genes between the normal and HCC samples was identified by building a difference comparison matrix. A heatmap and volcano map showed 47 downregulated genes and 35 upregulated genes (Figures 1A and B). The protein–protein interaction (PPI) network and co-expression among these genes are presented in Figure 1C.

### Functional enrichment and pathway analyses

**FIGURE 2:**
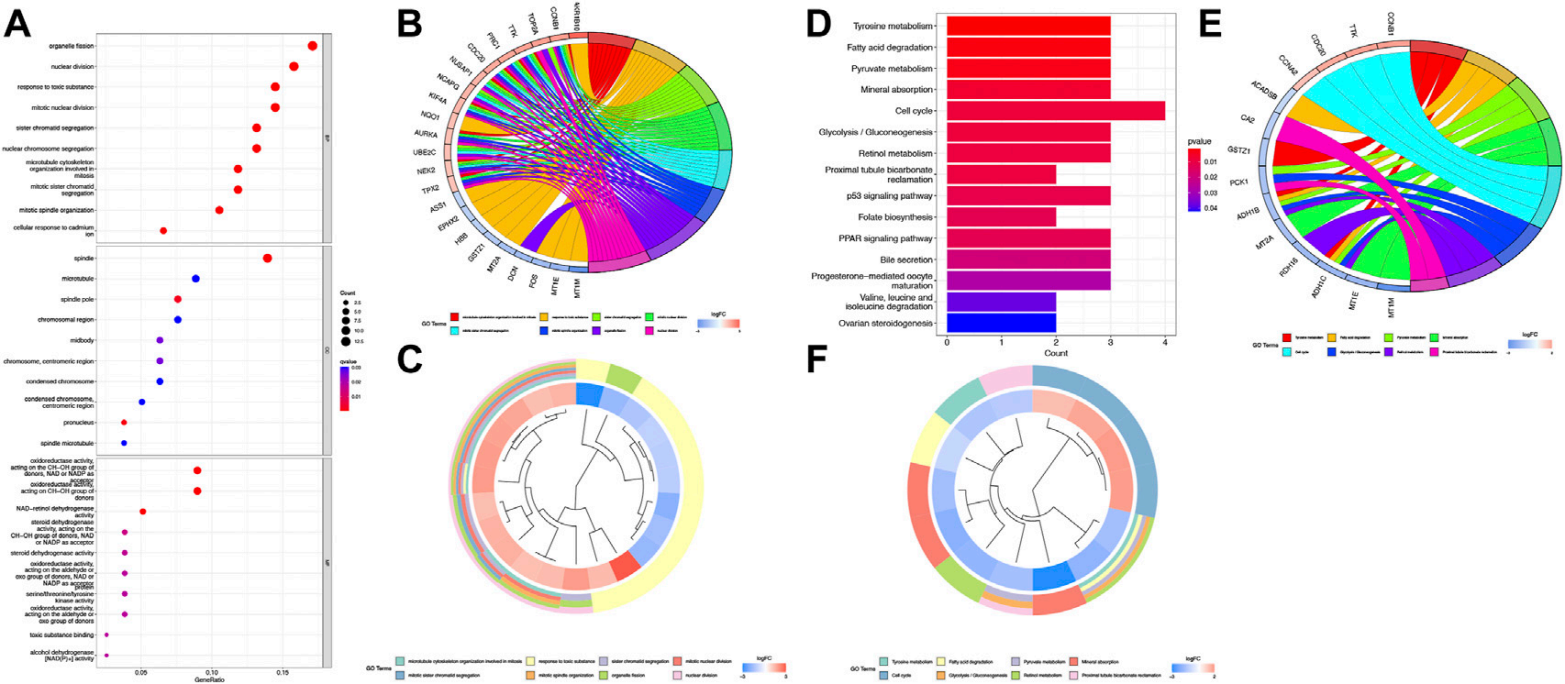
Function analysis based on the DEGs **(A)** Heatmap of GO analysis. **(B)** Circos plot of GO terms based on DEGs. **(C)** Cluster profiler analysis of the GO based on DEGs. **(D)** Barplot of KEGG terms based on DEGs. **(E)** Circos plot of KEGG terms based on DEGs. **(F)** Cluster profiler analysis of the KEGG based on DEGs.

KEGG and GO function enrichment analyses were performed here. As for GO function analysis, three GO terms were selected: molecular function (MF), cellular component (CC), and biological process (BP). Expression analysis showed that DEGs had the most uniquely enriched terms for organelle fission, (mitotic) nuclear division, and spindle, which were related to cell proliferation, cell division, and cell cycling. The processes mentioned were substantially over-represented during cancer transformation. Also, “oxidoreductase activity, acting on the CH–CH group of donors” was enriched in the list (Figure 2A). In Figures 2B, C, several up-regulated DEGs were mainly enriched in processes of “nuclear division,” “organelle fission,” etc., while down-regulated ones were mainly gathered in “response to toxic substance”.

Furthermore, KEGG pathway enrichment analysis indicated that DEGs were significantly enriched in “Cell cycle”, “Tyrosine metabolism”, etc (Figure 2D). In Figures 2E and F, upregulated DEGs were mainly enriched in the “Cell cycle” while downregulated DEGs were gathered in several cancer-related metabolism pathways.

**FIGURE 3:**
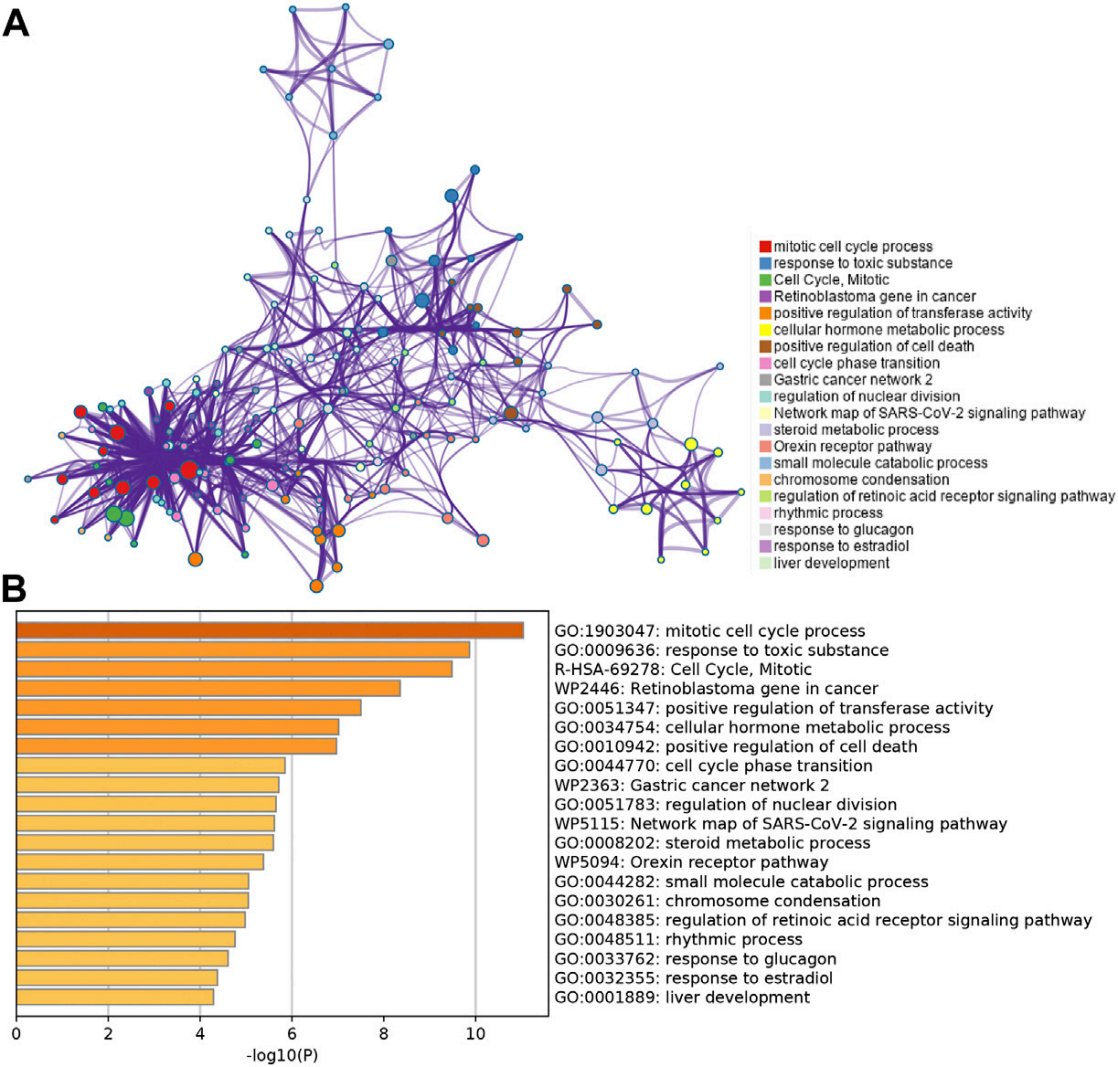
Functional pathway analysis using Metascape. **(A)** Network of enriched terms colored by the cluster identified in DEGs using the Metascape tool. **(B)** Top 20 clusters of enriched biological processes identified in DEGs using the Metascape tool.

To further validate and organize the results of KEGG and GO function, the DEGs were functionally annotated using Metascape. Metascape analysis showed the top 20 clusters of enriched biological processes like “mitotic cell cycle process,” “response to toxic substance,” etc (Figures 3A and B).

### Identification and validation of diagnostic-related genes

**FIGURE 4:**
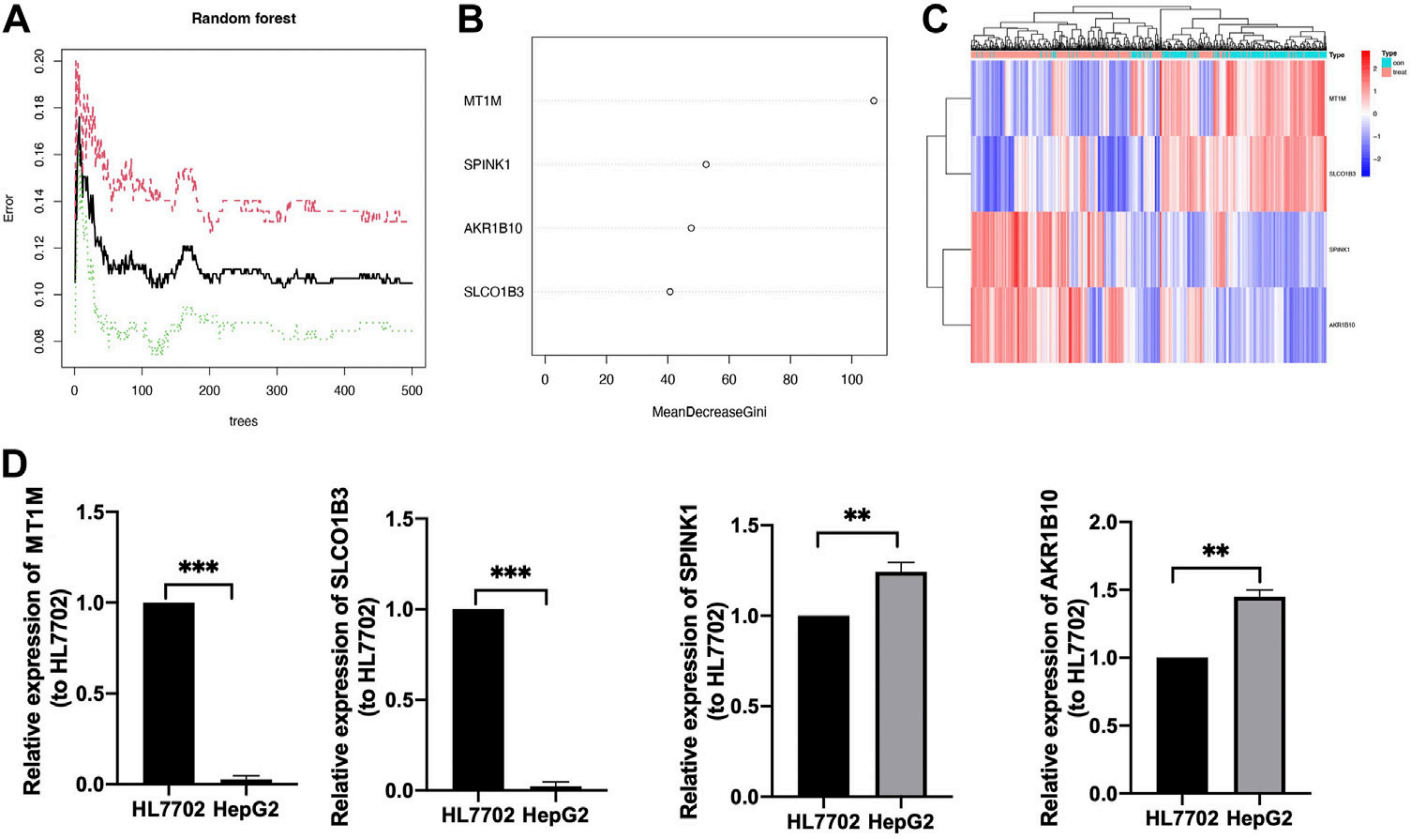
Identification of target genes. **(A)** Decision tree random forest tree. **(B)** Mean decrease in Gini coefficient of four target genes. **(C)** Heatmap of diagnostic candidate DEGs **(D)** QRT-PCR of four target genes, ^**^ *p* < 0.01, ^***^ *p* < 0.001.

Further comparison analysis between liver tissues from HCC and normal samples was identified by improving the screening criteria of logFC into |log_2_FC|>2, and we got four genes as candidate genes (Supplementary Figure S1). Next, a random forest analysis was performed, and four diagnostic-related genes (*MT1M*, *SPINK1*, *AKR1B10*, and *SLCO1B3*) were ensured, which showed that two genes were upregulated and two genes were downregulated (Figures 4A, B). The mean decrease in the Gini coefficient was a measure of how each variable contributed to the homogeneity of the nodes in the resulting random forest. The values were all over 40, which meant the four genes were of great importance in the development of the ANN model (Figure 4B). The diagnostic-related genes identified were shown in heatmap and could divide the training cohort into two groups (Figure 4C). Further validation of the diagnostic-related genes was performed by QRT-PCR (Figure 4D).

### Construction and validation of the diagnostic model built on an artificial neural network

**FIGURE 5:**
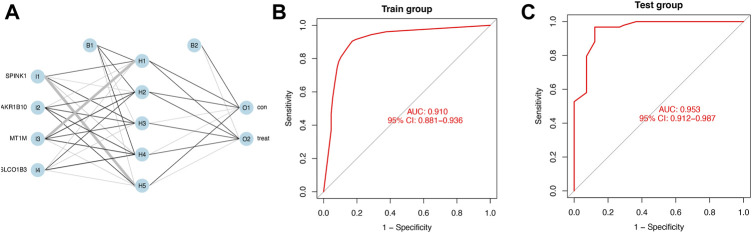
Diagnostic model constructed by the ANN **(A)** Schematic representation of the ANN model developed to predict the risk of HCC and normal samples. Thin lines represented synaptic weight <0; the thicker lines represented synaptic weight >0. **(B)** AUC of ROC curves verified the diagnostic performance of the ANN model in the training cohort. **(C)** AUC of ROC curves verified the diagnostic performance of the ANN model in the testing cohort.

We calculated the risk gene scores of the four genes in each sample to get the median cutoff value and defined the upregulated gene as “1” and the downregulated gene as “0” in each sample in the training and testing cohorts (Supplementary Table S2). The approximate ratio of the sample number in the training cohort (Normal:221; HCC:284) and the testing cohort (Normal:41; HCC:93) is 4 to 1. Then, the ANN method was performed to construct a diagnostic model based on gene scores of *MT1M, SPINK1, AKR1B10*, *and SLCO1B3* in each sample. The ANN model included three layers (input, hidden, and output) in which the number of nodes in the input layer and output layer was equal to 4 (number of input genes) and 2 (control and treatment), respectively (Figure 5A). An ROC curve was performed to detect whether the model could distinguish the HCC sample from the normal sample, and the area under the curve (AUC) was 0.910 (Figure 5B). In addition, the model worked well in the testing cohort as the AUC of ROC was 0.953 (Figure 5C).

### Infiltrating immune cell analysis

**FIGURE 6:**
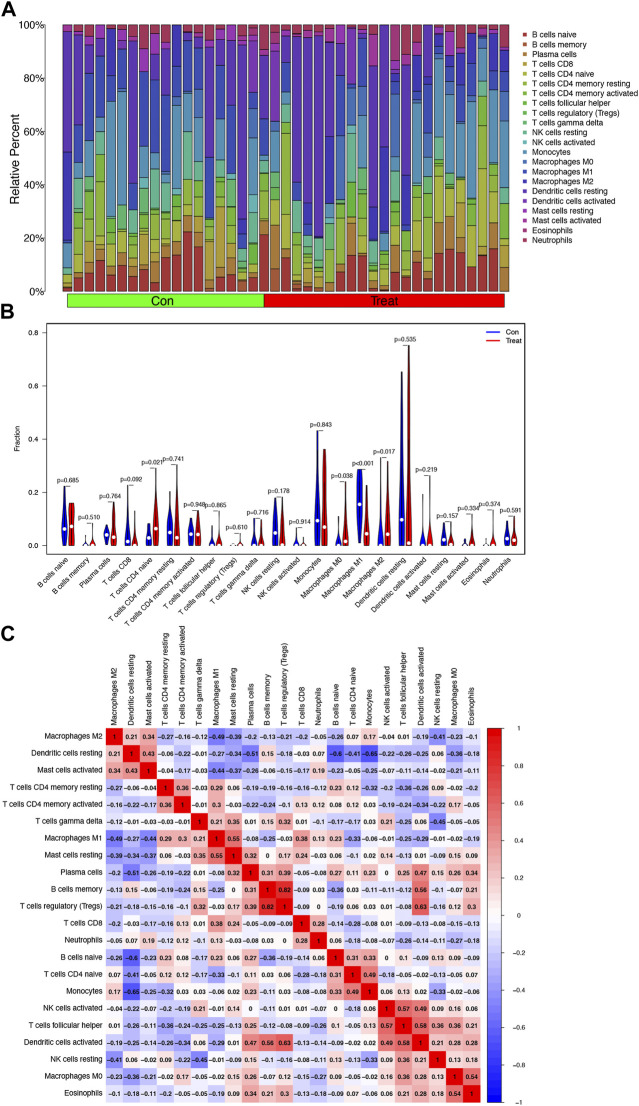
Analysis of infiltrating immune cells in the training cohort. **(A)** Heatmap of relative fraction of 22 representative immune cell population in the Con (normal) and Treat (HCC) cohorts was displayed. **(B)** Boxplots showed the cores of 22 immune cells between the Con (normal) and Treat (tumor) cohorts. **(C)** Heatmap of correlation between 22 immune cells.

The relative percent of 22 representative immune cells in the normal and HCC samples is presented in Figure 6A to show the approximate change in the proportion of immune cells in the training cohort. In Figure 6B, only “T cells CD4 naive”, “Macrophages M0”, “Macrophages M1”, and “Macrophages M2” displayed a substantial difference between the two groups (Figure 6B). “Macrophages M1” was the most significant one (*p* < 0.001) compared to “T cells CD4 naïve” (*p* = 0.021), “Macrophages M0” (*p* = 0.038), and “Macrophages M2” (*p* = 0.017). Furthermore, Macrophages M2 showed a significant negative correlation with Macrophages M1 and M0, while Macrophages M0 and M2 were both upregulated in the HCC group (Figures 6B, C).

### The sensitivity of diagnostic-related gene expression to presentative chemotherapy drug

**FIGURE 7:**
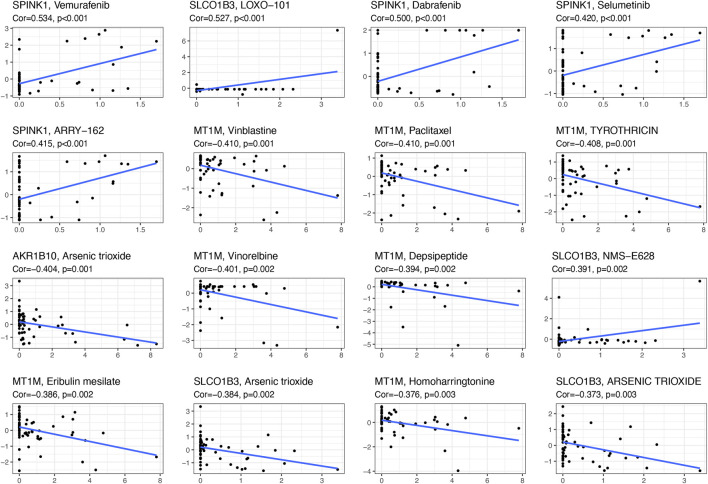
Scatter plot of the relationship between diagnostic-related gene expression and drug sensitivity.

With the help of NCI-60, a public database of human cancer cell lines, we determined the relationship between these diagnostic genes and drug sensitivity and showed the top 16 correlation analyses according to the *p*-value. Figure 7 demonstrated that *SPINK1* was sensitive to vemurafenib, dabrafenib, selumetinib, ARRY-162 (binimetinib) (*p* < 0.001), and *SLCO1B3* was sensitive to LOXO-101(larotrectinib) (*p* < 0.001) and NMS-E628 (*p* = 0.002), while it was insensitive to arsenic trioxide. In addition, the expression of *MT1M* was insensitive to vinblastine, paclitaxel, and tyrothricin (*p* < 0.001). Moreover, *AKR1B10* was insensitive to arsenic trioxide (*p* = 0.001).

### The cell viability under various presentative chemotherapy agents

**FIGURE 8:**
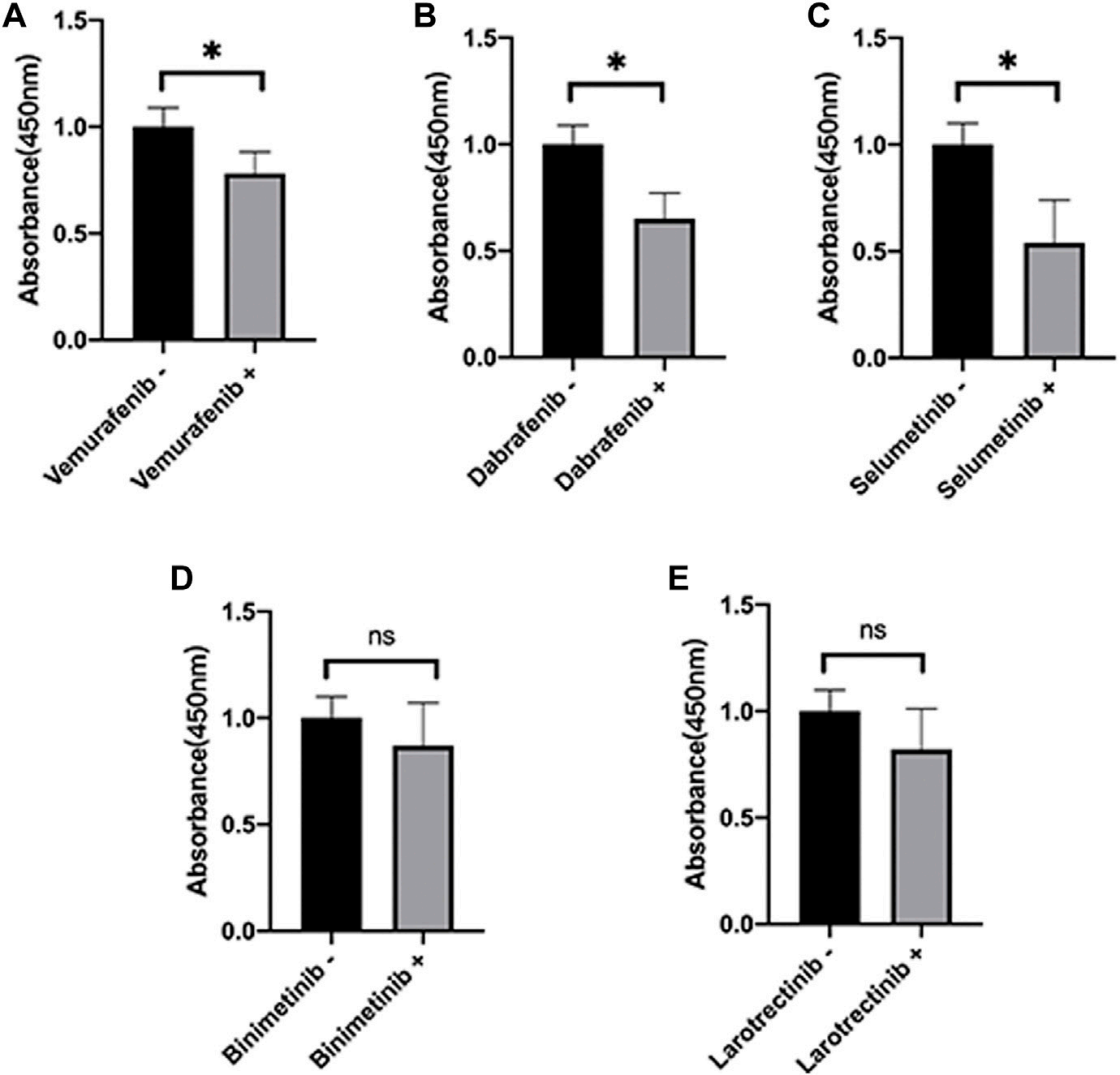
Cell viability of HepG2 under various presentative chemotherapy agent treatment in 24 h. The absorbance of HepG2 under treatment of **(A)** vemurafenib (5 μM), **(B)** dabrafenib (5 μM), **(C)** selumetinib (5 μM), **(D)** binimetinib (5 μM), and **(E)** larotrectinib (5 μM). ^*^ *p* < 0.05, ns = not significant.

Here, we further validate the HepG2 under various presentative chemotherapy drugs, including vemurafenib, dabrafenib, selumetinib, binimetinib, and larotrectinib, for they were sensitive to *SPINK1* and *SLCO1B3.* We found that the former three kinds of drugs could significantly inhibit the cell viability of HepG2, while the latter two could also work slightly without significant differences observed (Figure 8).

## Discussion

HCC is one of the most widespread problems facing by society today, which still has a high mortality rate in China (Xie et al., 20192020). Although comprehensive treatments have been adopted, HCC is associated with poor OS due to late diagnosis and high metastasis rate (Liu et al., 2020; Zhang et al., 2020). Early diagnosis, reasonable assessment of prognosis, and timely intervention are important for HCC patients, which encouraged us to explore better relevant biomarkers and diagnostic models (Beumer et al., 2021). Advancing molecular biology research methods brought diagnostic evaluation based on new types of biomarkers into reality. In this study, we observed 82 DEGs between HCC and normal samples. Based on DEGs, we explored the functional enrichment and pathway analyses and found that they were likely to be involved in mitosis and oxidative stress, which is consistent with the current latest research about cancer proliferation, metastasis, and treatment resistance. Moreover, in the infiltrating immune cell analysis, the unbalance of Macrophage M1/M2 was observed in this study. Macrophage M1 exerted cytotoxic function and eliminated early HCC, while macrophage M2 exerted anti-inflammatory activities and promoted cancer cell proliferation and invasion (Tian et al., 2019). However, Macrophage M0 and Naive T cell amounts were upregulated in the HCC cohort, while not all of them would differentiate to maturity and interfere with HCC proliferation or immigration. Thus, increasing the ratio of M1/M2 and the number of mature T cells might be a potential treatment for HCC (Dou et al., 2019; Yan et al., 2021).

To better select the candidate gene from DEGs for diagnostic model construction, we employed a random forest algorithm and found *MT1M*, *SLCO1B3*, *SPINK1*, and *AKR1B10* were the chosen ones. The mean decrease in the Gini coefficient of the four target genes was all above 40, which meant they had obvious specificity in DEGs. *MT1M*, *SLCO1B3*, *SPINK1*, and *AKR1B10* were cancer-related genes that were associated with different human diseases, especially in HCC. The specific biological functions of the four diagnostic-related genes (*MT1M*, *SLCO1B3*, *SPINK1*, and *AKR1B10*) in HCC in the recent 10 years are presented in Table 1.

**TABLE 1:** Various biological functions of four diagnostic-related genes in HCC.

Gene	Biological function	References
MT1M	Inhibiting proliferation, migration, invasion, and inducing apoptosis as well in HepG2 and Hep3B	(Changjun et al., 2018; Zhang et al., 2018)
	Promoter methylation of it could be regarded as serum biomarkers for noninvasive detection of HCC.	Ji et al. (2014)
SLCO1B3	It participated in drug absorption, distribution, metabolism, and excretion and was downregulated in HCC patients	Hu et al. (2019)
	Low expression of it might be a potential diagnostic, prognostic marker, targeted treatment in HCC patients and multistep hepatocarcinogenesis	(Yamashita et al., 2014; Chen et al., 2020; Kitao et al., 2020)
	However, SLCO1B3-mediated up-taking of indocyanine green was essential for HCC resection. It might also be related to poor prognosis of specific subclass of Wnt/β-catenin-activated HCC.	(Ueno et al., 2014; Shibasaki et al., 2015)
SPINK1	Promoting HCC cell proliferation, cell cycle, and invasion *in vitro*	(Huang et al., 2021; Lin et al., 2021)
	Downregulating E-cadherin and inducing EMT of HCC to promote metastasis	Ying et al. (2017)
	It could be regarded as a potential biomarker for early detection and targeted therapy of HCC.	(Marshall et al., 2013; Li et al., 2015; Jia et al., 2022)
	It was a downstream effector of the CDH17/β-catenin axis in HCC.	Shek et al. (2017)
AKR1B10	It might be a potential diagnostic biomarker for HCC development, metastasis, and a target for HCC-directed drug development	(DiStefano and Davis, 2019; Zhu et al., 2019)
		(Han et al., 2018; Ye et al., 2019)
	Inhibiting AKR1B10 expression elevated sorafenib’s anti-HCC effects via blocking the mTOR pathway, leading apoptosis and autophagy in HCC	Geng et al. (2020)
	It participated in the IRAK4/IRAK1/AP-1/AKR1B10 signaling pathway and AUF1-mediated post-transcriptional regulation of AKR1B10 expression to regulate cancer stemness and drug resistance in HCC.	(Cheng et al., 2018; Zhang et al., 2022)
	AKR1B10 expression was downregulated by fidarestat in NK cells, which promoted NK cell glycolysis to enhance killing ability to fight against HCC cells	Wu et al. (2021)
	The alteration rate of it increased significantly with the age of HCC patients	Atyah et al. (2018)
	Meanwhile, it played an important role in protecting hepatocytes from damage induced by ROS.	Liu et al. (2019)

In this study, we found the four target genes could divide the training cohort into two groups and have the same trend as in previous research. We also confirmed the expression trend of the four genes in HepG2 and HL7702 cell lines by QRT-PCR. Based on the four diagnostic-related genes, the ANN diagnostic model was developed and validated in GEO datasets. The ANN model obtained the highest prediction performance and has been widely used in various diseases to predict the population with high risk (Kourou et al., 2015; Zhong et al., 2019; Li et al., 2020). Based on the four diagnostic candidate genes, we successfully established a diagnostic model as for AUC of ROC was 0.910 and 0.953 in the training and testing cohorts, respectively, which meant it served as a reliable prediction model in our study.

According to the four diagnostic-related genes, we screened the potential drug that has a connection with diagnostic genes by NCI-60. We found that *SPINK1* was sensitive to vemurafenib, dabrafenib, selumetinib, and ARRY-162, and *SLCO1B3* was sensitive to LOXO-101 and NMS-E628. In addition, we further validated the cell viability of HepG2 under various presentative chemotherapy drugs, including vemurafenib, dabrafenib, selumetinib, binimetinib, and larotrectinib and observed vemurafenib, dabrafenib, and selumetinib might have a broad application prospect in HCC. Vemurafenib was a small-molecule inhibitor of the oncogenic v-raf murine sarcoma viral oncogene homolog B (BRAF) kinase that was used for treatment of melanoma (Hyman et al., 2015). However, increased *SPINK1* secretion was reported to be related to vemurafenib resistance in BRAF V600E-mutant colorectal cancers, indicating the need to target different gene variant subtypes of HCC during chemotherapy (29193645). Vemurafenib was also noticed to be the substrate of *SLCO1B3,* which might influence the absorption and elimination of the HCC chemotherapy drug (23340295). Nevertheless, BRAF gene polymorphisms were associated with capsule formation in HCC (Sun et al., 2021). BRAF-mutation-mediated MAPK pathway downstream was often constitutively activated and led to cancer cell differentiation, proliferation, angiogenesis, and anti-apoptosis, suggesting targeting the BRAF pathway might inhibit HCC progression in the future (Gnoni et al., 2019). Dabrafenib was also a selective inhibitor of BRAF kinase for patients suffering from BRAF-mutated melanoma, advanced non-small cell lung cancer, and anaplastic thyroid cancer harboring the BRAF^V600E^ mutation (Puszkiel et al., 2019). Until now, no association between dabrafenib and expression of *SPINK1* was reported in cancer treatment. Also, dabrafenib was found to inhibit the activation of OATP1B3 (*SLCO1B3*), which might contribute to increasing OATP1B3-substrate-sensitive drug during the absorption phase (Nebot et al., 2021). Considering the pharmacological mechanisms of dabrafenib and vemurafenib were similar, we also expected that dabrafenib might have prospects in the treatment of HCC. Selumetinib was a mitogen-activated protein kinase 1 and 2 (MEK1/2) inhibitor for treatment of neurofibromatosis, pediatric low-grade glioma, non-small cell lung cancer, and melanoma (Campagne et al., 2021). Selumetinib could be delivered by a novel delivery nanosystem in HCC and showed a well-targeted therapeutic strategy for HCC (Farinha et al., 2021). In addition, a combination of sorafenib and selumetinib could inhibit the growth of naïve and sorafenib-resistant HCC tumors via suppression of *β*-catenin signaling (Huynh et al., 2019). No specific research reported the direct connection among selumetinib, *SPINK1*, and *SLCO1B3.* However, *SPINK1* promoted HCC metastasis *via* the MEK/ERK signaling pathway (Ying et al., 2017). Similar research also reported that *SPINK1* expression in HCC cells was associated with HCC *via* activating the c-Raf/MEK/ERK pathway, which suggested that inhibiting the MEK pathway and usage of selumetinib could be a potential treatment strategy for HCC(37). In addition, the potential chemotherapy drug mentioned above mainly participated in cell proliferation, cell division, and cell cycling, which was consistent with our functional analysis and has not been fully used for HCC treatment in clinics (Davies et al., 2002; Yuan et al., 2020). Moreover, combined inhibition of BRAF and CSF-1R, which recruits M2-polarized macrophages in a tumor, resulted in superior antitumor responses (Mok et al., 2015). Although the potential drugs for chemotherapy were with broad application foreground, the diagnostic genes not only could enhance the drug sensitivity but also increased the resistance of chemotherapy drugs approved by the Food and Drug Administration (FDA). Thus, further research is needed for accurate application of these drugs in HCC.

## Conclusion

In conclusion, we constructed a novel diagnostic model based on four genes by the ANN model to predict the diagnosis of HCC patients. The model could provide useful insights into the potential prediction of HCC diagnosis. Several potential chemotherapy drugs came into view, although further research is required.

## Data Availability

The original contributions presented in the study are included in the article/Supplementary Material; further inquiries can be directed to the corresponding authors.
