# Supplementary figures and images for: A novel diagnostic four-gene signature for hepatocellular carcinoma based on artificial neural network: Development, validation, and drug screening
Source: Front Genet. 2022 Sep 28;13:942166. doi: 10.3389/fgene.2022.942166 (PMC9554094; doi:10.3389/fgene.2022.942166)

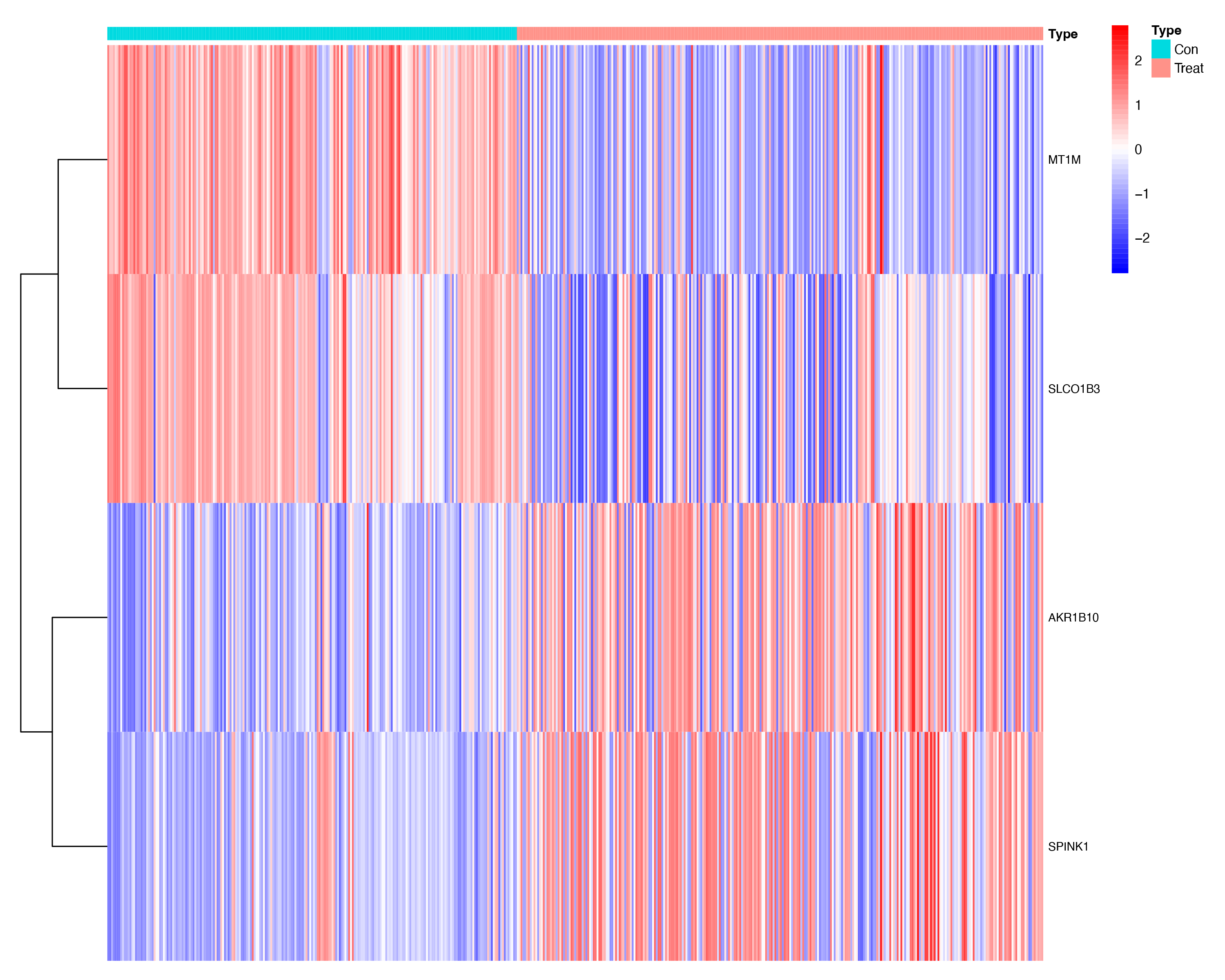

Supplement: Supplementary file 1 [file DataSheet1.ZIP › Supplementary/Figure S1.tif]
